# Gilbert White: Medical and Social Aspects of His Letters

**Published:** 1984-04

**Authors:** Philip Radford

**Affiliations:** Retired General Practitioner


### Gilbert White: Medical and Social Aspects of his Letters

**Philip Radford, M.B., Ch.B., D.C.H.**
Retired General Practitioner

Gilbert White, once curate of Selborne in Hampshire, is rightly famed for his classic descriptions of bird, mammal and insect behaviour and status in the eighteenth century, and immortalised in *The Natural History of Selborne*. Perhaps not so well known, however, is his interest in the public health of his parish, his respect for the individual, even if eccentric, and his sense of compassion. Furthermore, he was also concerned with diseases, remedies and superstitions, as they affected the inhabitants of Selborne.

Turning first to the public health of Selborne, Gilbert White noted 136 families, with about five persons per village tenement. The inhabitants were "sober and industrious" and lived in good stone or brick cottages which were glazed and had rooms above stairs; no mud buildings were utilised. For the 60 years 1720–79 there were 980 baptisms, of which 515 were males and 465 were females. Burials for the same period were 640, of which 315 were males and 325 females. Life expectation for men and women was equally good.

Twins occurred 13 times during the same period of time; many of these are reported as dying young, so White was aware of the greater infant mortality in multiple births associated, no doubt, with low birth weights. In general, White maintained that the inhabitants enjoyed "a good share of health and longevity" and the parish appeared to swarm with children.

Gilbert White considered country remedies important. So, when a quack at his village ate a toad "to make the people stare", he stated that oil was drunk after the strange meal. Further, common salad-oil was advocated as a remedy for the bite of a viper *Vipera berus*, but whether it was used internally or locally is not mentioned.

White could be sceptical in his assessment of a fashionable remedy and was so when it was asserted that toads could be used to cure cancer. It seems that a female healer at Hungerford claimed that, having "a virulent cancer", she was told by a strange clergyman that an application of living toads would cure her and, apparently, so it proved. Yet Gilbert White asks, logically, whether it was likely that a man with such knowledge would be prepared to treat one sufferer and to ignore all the others? Nevertheless, a fellow clergyman, with whom White dined, was able both to believe in and to approve of the treatment.

When White observed the behaviour of birds, he sometimes enquired if there was an analogy with the activities of man. Hence, when he noted the flocking together of birds, particularly in cold weather, he asked if this was related to the fact that men crowd together, when under "great calamities", although they do not know the reason. Of course, there is always the fear of a common enemy; maybe the avian flock makes the individual feel safer from a bird-of-prey.

Again, Gilbert White was willing to learn from the observations of others. He accepted the anatomical fact that nerves of significant size supply the upper mandible of rooks *Corvus frugilegus*. In consequence, it may be deduced that rooks have sensitive bills and so, by touch, are able to locate earthworms and other invertebrates in the soil.

Mr. White was prepared to examine the reasons behind the superstitions of his parishoners. For example, when he spoke of "a whole village up in arms . . . imagining the church-yard to be full of goblins and spectres", he explained how white owls *Tyto alba*, or barn owls as we would describe them today, produced their varied snoring, hissing or screaming noises and of the effect which these sounds had on the local people. Moreover, White mentioned that some country dwellers superstitiously believed that a screech-owl attends the windows of dying persons.

The reeling sounds of the song of the grasshopper warbler *Locustella naevia* intrigued Gilbert White as might have been expected; showing a sense of humour, he did not mind admitting that the country people laughed when told that the sound was made by a bird. Clearly, he took notice of the beliefs of his neighbours, as well as paying attention to old rural sayings. In one of his letters, for instance, he told how country people said that when land-springs rise, corn will be dear: cereals cannot grow well in flooded earth. Philosophically, the curate remarked ". . . . we must not expect plenty till Providence sends us more favourable seasons".

At times, Gilbert White became angry, as when a "booby of a carter" threw down two white rooks from their nest. Again, when referring to swifts *Apus*

*apus*, White remarks that they are "wantonly and cruelly shot while they have young . . ." and he evidently regretted the fact that swifts were often "beaten down with poles and cudgels as they stoop to go under the eaves". Although Gilbert White approved of the shooting of birds for the purpose of identification, he disliked any unnecessary cruelty.

White does not ignore obstetrics and, when remarking on the hardiness of gypsies, he described how a young gypsy girl was delivered of her baby in the middle of a hop-garden. Apparently, she had a covering of a blanket looped over hazel-rods but disdained the shelter of a large hop kiln during very wet September weather. Referring to his own ailments, White stated in 1774 that ". . . returns of deafness incommode me sadly" although, happily, he was able to admit that his eyesight at that time was ". . . quick and good".

Domestic economy was considered an important topic, with White discussing the preparation of rush-lights; costing showed that a family could obtain $5\frac{1}{2}$ hours of light for one farthing. Then White was of the opinion that the very poor were the worst economists where lighting was concerned, for they would buy one halfpenny candle each evening and this would only burn for two hours.

White did not forget the mentally disabled. He tells how an "idiot-boy" just lived for bees; in the summer the boy would catch hive-bees, bumble bees and wasps. The sting would be removed and the insects were sucked for their honey content; furthermore, the boy would sit by hives and grasp bees as they emerged. He would also hum and try to imitate the buzzing of bees. This is an example of a person who became skilled at one subject but otherwise showed "no manner of understanding"; he died "before he arrived at manhood".

Again discussing superstition and prejudice, White recalled that as late as 1751, at Tring in Hertfordshire, two old women were drowned in a pond on suspicion of witchcraft. Then, near Selborne it was the custom for ruptured children to be pushed through cleft ash trunks; the children were naked during the procedure and, when it was over, the cleft tree was plastered and tied. Hernias were said to have been cured when the split trees became sealed over!

The effects of castration were understood by White and this was not only amongst farm animals. Thus, human eunuchs were noted to have high-pitched voices and to be beardless. Changes in behaviour were also seen to occur if horns or tusks were damaged in farm stock. A fierce boar, for instance, which had its tusks broken, became quite placid as a result; one is reminded that Samson lost power when his hair was cut off!

The physical effects of sudden climatic change were not overlooked. Apparently, in 1777 two very hot days occurred at the end of March and, as a result of the sultry conditions, people complained of restlessness. According to White, these extreme temperatures were followed by abnormal spring weather, with strong winds together with frosts.

Returning to medical matters, White told of a pauper who was afflicted by "a leprosy" which affected only the palms of his hands and the soles of his feet. A scaly eruption occurred twice a year, leaving the skin so tender that the patient was obliged to use crutches. He died, incapable of employment and supported by the parish at "more than thirty years of age". One cannot but wonder if the true diagnosis was a pustular psoriasis.

However that may be, writing in 1778, White considered the reasons why leprosy had declined in incidence, so that leper hospitals were no longer required. He thought that improved nutrition was one factor; previously, salted fish or meat only could have been obtained in winter but, latterly, with the improved feeding of cattle, fresh meat could be bought in winter. Further, better bread of wheat origin became available, together with fresh produce such as fruit, beans and peas, greens and root crops.

Then clothing had altered, with linen replacing wool as the material in contact with the skin. It seems that, at Selborne at least, labourers had gardens to grow their own crops and potatoes were beginning to get popular as food. Doubtless, improved diet and clothing was of value in reducing skin disorders in general; certainly avitaminosis could be prevented and the use of linen would have helped in cutting the incidence of cutaneous infestations and sensitisation reactions. Of course, in White's day, any skin eruption with silvery scales was thought of as leprosy and, no doubt, many sufferes from psoriasis had quite unnecessary restrictions imposed on them.

Understandably, herbal remedies are mentioned in the letters and there is a reference to the hellebore *Helleborus foetidus*: it seems that the "good women give the leaves powdered to children troubled with worms; but it is a violent remedy, and ought to be administered with caution".

As with his exact and meticulous natural history observations, Gilbert White took notice of all human activity in his neighbourhood. In consequence, we are left with a unique, reliable and often detailed account of the social, economic and medical events of Selborne in the eighteenth century. Gilbert White was a man searching for truth; facts were recorded without bias, but usually his views and opinions were tempered with humanity and sympathy.

### REFERENCES

WHITE, G. (1789) The Natural History and Antiquities of Selborne, in the County of Southampton. London.

